# Supplementary material for: MRI-Based Radiomics Differentiates Skull Base Chordoma and Chondrosarcoma: A Preliminary Study
Source: Cancers (Basel). 2022 Jul 3;14(13):3264. doi: 10.3390/cancers14133264 (PMC9265125; doi:10.3390/cancers14133264)
Supplement: Supplementary file 1 [file cancers-14-03264-s001.zip › cancers-1740647-supplementary.pdf]

Supplementary Materials: MRI-Based Radiomics Differentiates Skull Base Chordoma and Chondrosarcoma: A Preliminary Study

Erika Yamazawa, Satoshi Takahashi, Masahiro Shin, Shota Tanaka, Wataru Takahashi, Takahiro Nakamoto, Yuichi Suzuki, Hirokazu Takami and Nobuhito Saito

Some of our scripts are available on GITHUB below.

https://github.com/satoc1907/Radiomics_Chordoma_and_Chondrosarcoma

Table S1. Imaging parameters of MRI systems

**GdT1**	**MR1**	GE, Signa HDxt, 1.5T, Repetition time/echo time (TR/TE) =9.8ms/3.9ms, slices = 128, acquisition matrix =256 ×256, Field Of View (FOV) =240mm3, Flip angle=20 degree, inversion time= 400ms, slice thickness 1.5mm, acquisition time = 476 s
	**MR2**	SIEMENS MAGNETOM Skyra, 3T, TR/TE =1910ms/3.25ms, slices = 192, acquisition matrix =256 ×256, FOV=240mm3, Flip angle=8 degree, inversion time= 962ms, slice thickness 1.0mm, acquisition time = 236s
	**MR3**	GE SignaHDxt, 3T, TR/TE =6.9ms/2.6ms, slices = 200, acquisition matrix =256 ×256, FOV=240mm3, Flip angle=15 degree, inversion time= 450ms, slice thickness 1.0mm, acquisition time = 359s
	**MR4**	GE SignaPremier, 3T, TR/TE =6.2ms/2.5ms, slices = 200, acquisition matrix =256 ×256, FOV=240mm3, Flip angle=8 degree, inversion time= 900ms, slice thickness 1.0mm, acquisition time = 230s
**T2**	**MR1**	GE, Signa HDxt, 1.5T, Repetition time/echo time (TR/TE) =4000ms/105ms, slices = 23, acquisition matrix =256 ×256, Field Of View (FOV) =210mm3, Flip angle=90 degree, inversion time= NA, slice thickness 5.0mm, acquisition time = 112 s
	**MR2**	SIEMENS MAGNETOM Skyra, 3T, TR/TE =4000ms/84ms, slices = 50, acquisition matrix =384 ×307, FOV=210mm3, Flip angle=90 degree, inversion time= NA, slice thickness 3.0mm, acquisition time = 176s
	**MR3**	GE SignaHDxt, 3T, TR/TE =4360ms/85ms, slices = 50, acquisition matrix =384 ×256, FOV=210mm3, Flip angle=90 degree, inversion time= NA, slice thickness 3.0mm, acquisition time = 150s
	**MR4**	GE SignaPremier, 3T, TR/TE =4332ms/85ms, slices = 50, acquisition matrix =384 ×256, FOV=210mm3, Flip angle=90 degree, inversion time=NA, slice thickness 3.0mm, acquisition time = 148s
	**MR5**	Philips IngeniaCX, 3T, TR/TE =4165ms/90ms, slices = 50, acquisition matrix =384 ×265, FOV=210mm3, Flip angle=90 degree, inversion time= NA, slice thickness 3.0mm, acquisition time = 150s

Abbreviations: post-gadolinium T1-weighted images (GdT1), T2 weighted image (T2).

Table S2. Radiomics features obtained from images.

**Morphological featurs (shape/size) (8)**	Compactness1, Compactness2, Max3D diameter, Spherical Disproportion, Sphericity, Surface Area, Surface to volume ratio, Volume
**Intensity (18)**	Maximum intensity, Minimum intensity, Mean intensity, Median intensity, Intensity range, Intensity variance, Intensity skewness, Intensity kurtosis, Intensity-based energy, 10th intensity percentile, 90th intensity percentile, Intensity interquartile range, Intensity-based mean absolute deviation, Intensity-based robust mean absolute deviation, Intensity-based median absolute deviation, Intensity-based quartile coefficient of dispersion, Root mean square intensity, Intensity-based coefficient of variation
**Intensity histogram (20)**	Mean discretisedintensity, Discretisedintensity variance, Discretisedintensity skewness, Discretisedintensity kurtosis, Median discretisedintensity, 10th discretisedintensity percentile, 90th discretisedintensity percentile, Intensity histogram mode, Discretisedintensity interquartile range, Intensity histogram mean absolute deviation, Intensity histogram robust mean absolute deviation, Intensity histogram median absolute deviation, Intensity histogram coefficient of variation, Intensity histogram quartile coefficient of dispersion, Discretisedintensity entropy, Discretisedintensity uniformity, Maximum histogram gradient, Maximum histogram gradient intensity, Minimum histogram gradient, Minimum histogram gradient intensity
**Gray-level co-occurrence matrices (GLCMs) (11)**	Energy, Contrast, Correlation1, Correlation2, Inverse Difference, Inverse Difference Moment, Variance, Sum Average, Entropy, Dissimilarity, AutoCorrelation
**Gray-level run-length matrices (GLRLMs) (13)**	Short Run Emphasis, Long Run Emphasis, Gray-Level Non-uniformity, Run-Length Non-uniformity, Run Percentage, Low Gray-Level Run Emphasis, High Gray-Level Run Emphasis, Short Run Low Gray-Level Emphasis, Short Run High Gray-Level Emphasis, Long Run Low Gray-Level Emphasis, Long Run High Gray-Level Emphasis, Gray-Level Variance, Run-Length Variance
**Gray-level size-zone matrices (GLSZMs) (13)**	Small Zone Emphasis (SZE), Large Zone Emphasis (LZE), Gray-Level Non-uniformity (GLN), Zone-Size Non-uniformity (ZSN), Zone Percentage (ZP), Low Gray-Level Zone Emphasis (LGZE), High Gray-Level Zone Emphasis (HGZE), Small Zone Low Gray-Level Emphasis (SZLGE), Small Zone High Gray-Level Emphasis (SZHGE), Large Zone Low Gray-Level Emphasis (LZLGE), Large Zone High Gray-Level Emphasis (LZHGE), Gray-Level Variance (GLV), Zone-Size Variance (ZSV)
**Neighborhood grey-level different matrix (NGLDM) (16)**	Low dependence emphasis, High dependence emphasis, Low grey level count emphasis, High grey level count emphasis, Low

	dependence low grey level emphasis, Low dependence high grey level emphasis, High dependence low grey level emphasis, High dependence high grey level emphasis, Grey level non-uniformity, Normalised grey level non-uniformity, Dependence count non-uniformity, Dependence count percentage, Grey level variance, Dependence count variance, Dependence count entropy, Dependence count energy
**Neighborhood gray-tone difference matrices (NGTDMs) (5)**	Coarseness, Contrast, Busyness, Complexity, Strength

Numbers in parentheses indicate the numbers of radiomics features.

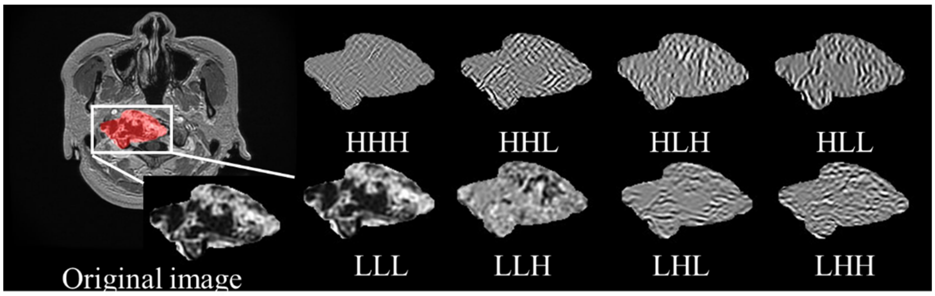

Figure S1. Wavelet transformation of the image. Volume Of Interests (VOIs) on the original images and 8 sub-bands filtered images using wavelet transform. "H" and "L" denote "high-pass" and "low-pass", respectively.

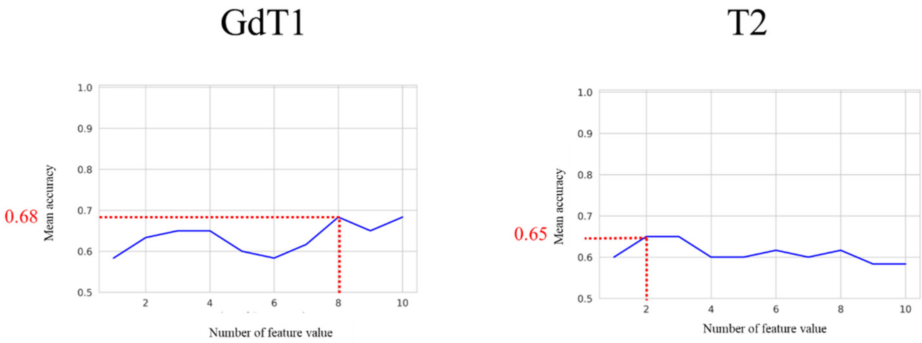

Figure S2. Optimal numbers of features**.** Optimal numbers of features for post-gadolinium T1-weighted images (GdT1) only model and T2 weighted images (T2) only model. The smallest number of features was selected when the mean accuracy was the same.
